# The Effect of a Third In-Ear Microphone on User Satisfaction, Speech Intelligibility, and the Real-Ear Gain of Hearing Aids at a Conversational Level in Patients with Moderate Hearing Loss

**DOI:** 10.3390/jcm14196791

**Published:** 2025-09-25

**Authors:** Sang Hyun Kwak, Dongchul Cheon, Seong Hoon Bae, Daeyoung Kim, Jinsei Jung

**Affiliations:** 1 Department of Otorhinolaryngology, St. Vincent Hospital, College of Medicine, The Catholic University of Korea, Seoul 06591, Republic of Korea; kwaksang84@gmail.com; 2 Department of Otorhinolaryngology, Yonsei University College of Medicine, Seoul 03722, Republic of Korea; paulbag@naver.com (D.C.); bshsap1@yuhs.ac (S.H.B.); 3 Won-Sang Lee Institute for Hearing Loss, Seoul 03722, Republic of Korea; 4 Research Center of GN Hearing Korea, Seoul 06236, Republic of Korea; rs_admin@resoundkr.com

**Keywords:** hearing loss, hearing aid, third microphone, real-ear gain, feedback problem

## Abstract

**Background:** The microphone & receiver-in-ear (M&RIE) integrates two traditional hearing aid microphones, while an additional in-ear microphone helps preserve natural sound perception. However, the impact of this third microphone on hearing aid amplification remains unclear in patients with moderate hearing loss. **Methods:** In this prospective crossover study, thirty-nine participants with moderate hearing loss and no prior hearing-aid use were randomly assigned to be sequentially fitted with both traditional and M&RIE receivers. The abbreviated profile of hearing aid benefit (APHAB) and word recognition score (WRS) were assessed. Audiological amplification was evaluated using real-ear measurements to determine whether a third in-ear microphone limits real-ear gain. **Results:** WRSs and APHAB scores showed no significant differences between the standard and M&RIE receivers. In addition, real-ear measurements across all frequencies and earplug types revealed no significant differences in real-ear insertion gain between the two receivers at a conversational level (65 dB SPL). **Conclusions:** The M&RIE provides amplification comparable to that of the standard receiver while preserving natural sound cues without significant audiological disadvantages.

## 1. Introduction

Hearing aids (HAs) have evolved significantly to enhance speech perception, spatial awareness, and overall sound quality in individuals with hearing loss. Traditional receiver-in-the-ear (RITE) HAs use externally placed microphones, which limit access to the natural pinna cues that are essential for sound localization and spatial hearing [1]. The auricle (pinna) and concha naturally boost high-frequency sounds, thereby facilitating sound-source localization. Although directional microphones are widely used to improve speech intelligibility in noisy environments, their effectiveness varies depending on the microphone placement and signal processing algorithms [2]. A comparative study of in-the-ear (ITE) and behind-the-ear (BTE) dual-microphone hearing aids demonstrated that microphone placement significantly affects speech clarity, localization, and subjective satisfaction [3]. In addition, the occlusion effect, a phenomenon in which low-frequency sounds are amplified due to ear canal blockage, is influenced by both the depth of receiver insertion and the design of the HA [4,5].

Recent research has shown that the microphone position can influence speech intelligibility, spatial perception, feedback resistance, occlusion effects, and user satisfaction [6,7,8]. For instance, a study comparing receiver-in-the-ear (RITE) and transducer-in-the-ear (TIE) designs reported that TIE configurations, with microphones positioned closer to the ear canal, provided superior speech recognition in noise, greater sound localization accuracy, and higher user satisfaction [1,9]. It has also been suggested that microphones placed at the ear canal entrance preserve natural spectral cues, thereby enhancing sound localization [10,11]. The perceived sound quality of HAs with different microphone and receiver placements indicates that users prefer devices offering a wider bandwidth, particularly beyond 5 kHz for speech and below 300 Hz for music [4].

When microphones are positioned behind the ear, natural acoustic modifications of the auricle and concha are often lost, resulting in challenges with front–back localization and sound externalization [6,10,12,13,14,15]. To address these limitations, microphone & receiver-in-the-ear (M&RIE) technologies have been introduced, incorporating a third microphone within the ear canal [16]. The strategic placement of this microphone allows the device to capture sound while preserving the pinna’s natural spectral shaping effects, thereby improving spatial hearing and sound localization accuracy [17]. In addition, because the third microphone is located inside the ear canal rather than behind the ear, it is less exposed to wind, which may reduce wind interference [18]. By leveraging an individual’s external auditory anatomy to filter sounds, the M&RIE technology enables users to perceive the direction and distance of sound sources more naturally. Notably, placement of the third microphone has also been shown to improve speech identification and perceived sound quality through the preservation of pinna and ear canal resonance [17].

Despite numerous advancements in M&RIE technology, limited clinical evidence exists regarding whether an M&RIE receiver can provide sufficient real-ear gain across a wide range of input signals and frequencies. Because the M&RIE receiver is positioned adjacent to the microphone in the ear, potential feedback issues may restrict amplification at certain frequencies. In particular, loud input sounds may limit the achievable amplification without causing subjective discomfort. Therefore, this study aimed to compare the performance of the M&RIE receiver with traditional receivers in terms of speech recognition, user satisfaction, and amplification. Particularly, we focused on whether the M&RIE receiver provides sufficient real-ear gain at a conversational level for individuals with moderate hearing loss. Our goal was to determine whether an M&RIE receiver can serve as a viable alternative to conventional HA designs. Establishing the effectiveness of this technology will help clinicians provide evidence-based recommendations and optimize HA fittings for individuals seeking improved spatial hearing, sound quality, and overall satisfaction.

## 2. Materials and Methods

### 2.1. Study Design

This single-center prospective study included 39 adults diagnosed with moderate sensorineural hearing loss (SNHL), as defined by pure tone audiometry (PTA), ranging from 40 to 70 dB HL. Based on an a priori power calculation (two-sided α = 0.05 and *n* = 39, paired design with each participant trialing both receivers), the study had 80% power to detect a Cohen’s dz of 0.45 (≈0.52 for 90% power). This study was adequately powered to detect moderate within-subject differences between receivers. All participants were prescribed HA for auditory rehabilitation and had no prior HA use. This study was conducted in accordance with the recommendations of the Ethics Committee of the Yonsei University (IRB number: 1-2021-0071). We confirmed that all protocols for human experiments were approved by the institutional review board of Yonsei University College of Medicine. All participants provided written informed consent, which was obtained at the timepoint of their enrolment in the study, and the legal guardians of all minors and children enrolled in this study provided consent for their words. This research was performed in accordance with the Declaration of Helsinki.

### 2.2. Randomization and Hearing Aid Fitting Protocol

**Figure 1:**
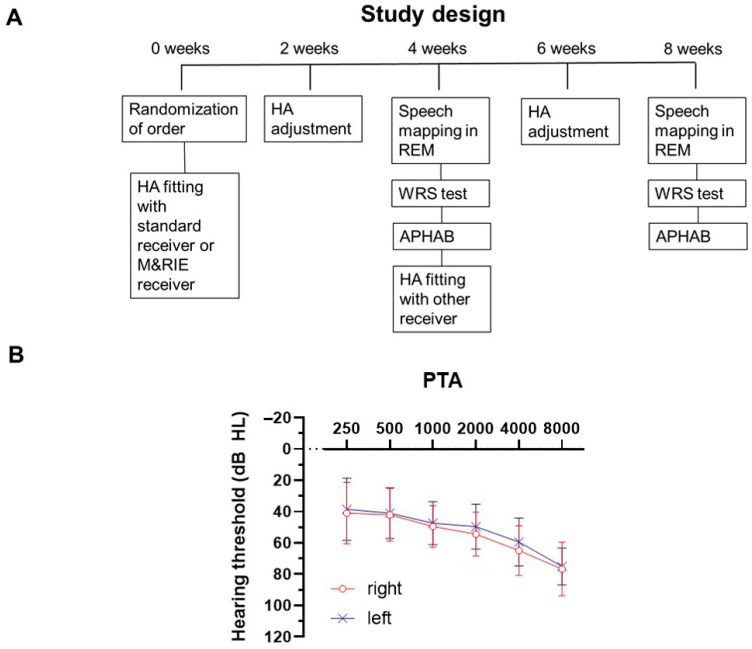
**Study Design Schematic and Participant Audiometric Profile.** (**A**) Schematic representation of the study protocol. Participants were randomly assigned to initially receive either the standard receiver or the Microphone & Receiver-in-Ear (M&RIE) receiver. A crossover design was implemented. After an initial 4-week trial period, hearing aid adjustments were made at 2 weeks, followed by assessments including speech mapping in real-ear measurement (REM), word recognition score (WRS) tests, and the Abbreviated Profile of Hearing Aid Benefit (APHAB) questionnaire. Participants then switched to the alternate receiver, with the same protocol repeated. (**B**) Pure Tone Audiometry (PTA_4_) results, displaying the average hearing thresholds across four key frequencies (500, 1000, 2000, and 4000 Hz) for all participants. The numbers of right and left ears are 37 and 32, respectively.

Participants were randomly assigned to one of the two groups to determine the initial order of receiver fitting–either the standard or M&RIE receiver. A crossover design was implemented to ensure that each participant experienced both receiver types during the study period. Figure 1A shows a schematic of the study protocol.

ReSound Omnia RU9 Receiver-In-Ear (RIE) HAs (GN Hearing, Ballerup, Denmark) were used. To determine the target gain, the initial fit for all participants was based on the NAL-NL2 formula. Participants used the assigned receiver (either standard or M&RIE) for 4 weeks. Additional adjustments were made after two weeks. At the initial fitting, real ear insertion gain (REIG) was targeted to the gain values prescribed by the NAL-NL2 formula, within a range of ±10 dB; however, subsequent fine-tuning was based on user preference. At the end of the first 4-week trial, participants underwent the following assessments: Speech mapping in real ear measurement (REM) was conducted to verify the REIG and ensure that the output remained consistent with the target gain (Aurical Freefit). A word recognition score (WRS) test was conducted to assess speech understanding in quiet conditions. Because this study aimed to evaluate the limitations of amplification in the M&RIE receiver, the WRS test was performed only in quiet conditions, without enabling additional noise-cancelation functions. The abbreviated profile of hearing aid benefit (APHAB) questionnaire was used to evaluate subjective satisfaction across various listening environments, including ease of communication, reverberation, and background noise.

### 2.3. Audiologic Assessment

Prior to HA fitting, all participants underwent comprehensive audiological evaluation, including PTA and speech audiometry, to diagnose and establish baseline auditory function as previously reported [19,20,21,22]. The average of four frequencies (500, 1000, 2000, and 4000 Hz) presented in a pure-tone audiogram (PTA_4_) format was calculated and defined as the mean value. The WRS test was conducted for speech audiometry in a quiet environment using a phonetically balanced word list presented at the participants’ most comfortable listening level (MCL) [23]. The WRS was expressed as the percentage of correctly identified words, providing an objective measure of speech discrimination ability. The MCL was determined by gradually increasing the intensity of speech stimuli until the participant indicated the most comfortable loudness level for listening as previously reported [24].

REM was performed to verify the accuracy of the HA fitting based on the NAL-NL2 formula. Speech mapping was used to determine whether the amplified output of the HA matched the target gain. A 65 dB SPL input signal was applied to measure REIG, as this level reflects typical real-world conversational environments. REIG was calculated as the difference between unaided and aided responses within the ear canal.

The APHAB questionnaire [20] was administered to assess subjective HA satisfaction across the following subscales: ease of communication (EC), the ability to understand speech in quiet environments; background noise (BN), difficulty understanding speech in noisy environments; reverberation (RV), difficulty understanding speech in echoic environments; and aversiveness (AV), discomfort caused by loud sounds.

### 2.4. Statistical Analysis

Statistical analyses were performed using Statistical Package for the Social Sciences version 22 for Windows (IBM Corp., Armonk, NY, USA) and GraphPad Prism version 5 (GraphPad Software, La Jolla, CA, USA). Audiological outcomes and APHAB were compared and analyzed using the paired Student’s *t*-test. Differences between the mean values of the groups were considered statistically significant at *p* < 0.05.

## 3. Results

### 3.1. Participant Demographics

A total of 39 adult participants with moderate SNHL were enrolled in this study (Table 1). A total of 69 patient ears were fitted with HAs. The mean PTA_4_ was 52.4 ± 11.1 dB HL at right ear and 48.9 ± 11.2 dB HL at left ear (Figure 1B). Participants expressed their preference for the receiver type based on their experience: in total, 23 participants (59.0%) preferred the standard receiver, and 16 participants (41.0%) preferred the M&RIE receiver.

### 3.2. Comparisons of APHAB Analysis

**Figure 2:**
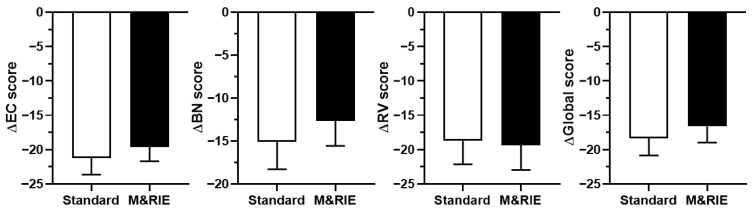
**Abbreviated Profile of Hearing Aid Benefit (APHAB) Scores for standard and M&RIE receivers.** This figure shows APHAB results from 39 participants, assessing subjective satisfaction across four subscales: Ease of Communication (EC), Background Noise (BN), Reverberation (RV), and Global score. No significant differences were observed between the standard and M&RIE receivers in any subscale (*p* > 0.05, paired *t*-test). The M&RIE receiver showed slight, non-significant trends toward improved performance in EC and BN.

Figure 2 presents the results of the APHAB questionnaire, which assessed participants’ subjective satisfaction across the four key subscales. Based on responses from 39 participants, the analysis revealed no significant differences between the standard and M&RIE receivers on any APHAB subscale. Both receiver types demonstrated comparable satisfaction levels in daily listening environments. Interestingly, although not statistically significant, the M&RIE receiver showed a slight trend toward better performance in the background noise subscale.

### 3.3. Comparisons of Speech Intelligibility

**Figure 3:**
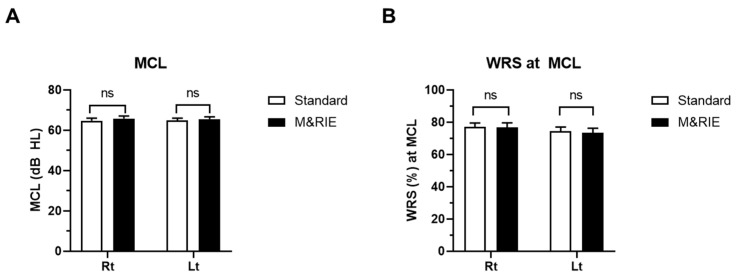
**Comparison of most comfortable listening Level (MCL) and word recognition score (WRS) between standard and M&RIE receivers.** (**A**) MCL reported by participants when using the standard and M&RIE receivers (*n* = 39 ears at each group). No significant differences were observed, indicating similar comfort across devices. (**B**) WRS assessed at each participant’s MCL (*n* = 39 ears at each group). Both receivers showed comparable speech recognition performance in quiet environments, with no statistically significant differences.

Figure 3 shows a comparison of MCL and WRS. There were no significant differences in MCL between the standard and M&RIE receivers. Participants reported similar loudness comfort levels across both devices (Figure 3A). WRS was also comparable between the two receiver types (Figure 3B). Both receivers demonstrated similar speech recognition performance under quiet conditions, with no statistically significant differences observed.

### 3.4. Comparisons of REIG Analysis

**Figure 4:**
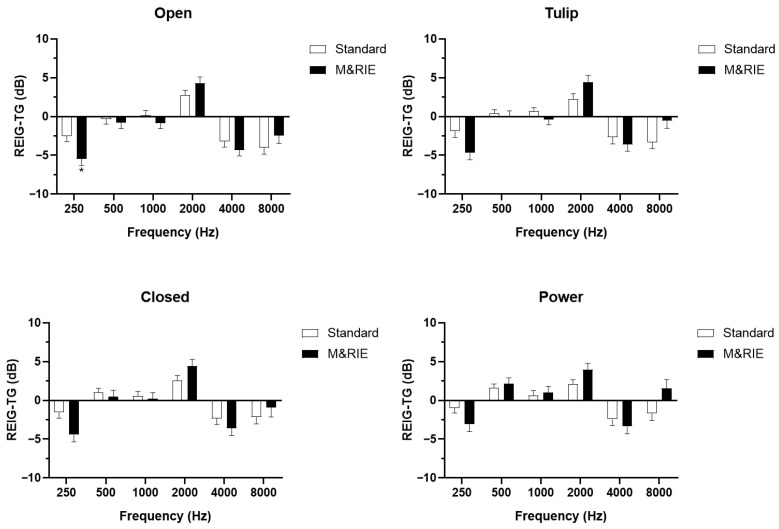
**Comparison of Real-Ear Insertion Gain (REIG) between standard and M&RIE receivers.** REIG was measured across 69 ears fitted with either the standard or M&RIE receivers. No significant differences in REIG values were observed between the two receiver types across all tested frequencies and dome types (*p* > 0.05, paired *t*-test). These results indicate that the M&RIE receiver provides amplification comparable to the standard receiver and effectively meets the NAL-NL2 prescribed target gains. A total of 69 ears were fitted with each dome type; open domes have large vents, closed domes have smaller or no vents, tulip domes use two overlapping flaps, and power domes have a double-layered structure that creates the tightest seal.

Figure 4 illustrates the comparison of REIG between the standard and M&RIE receivers across 69 ears. The analysis showed no significant differences in REIG values between the two receiver types, regardless of the earplug type, across all tested frequencies at a conversational level with 65 dB SPL input sound.

## 4. Discussion

This study evaluated the audiological performance and user satisfaction of M&RIE receivers compared to standard receivers in adults with moderate SNHL. The M&RIE receiver incorporates a third microphone within the ear canal to preserve the natural pinna cues, which are essential for sound localization and spatial awareness [4,17]. By leveraging the individual’s ear anatomy, this design enhances spatial hearing and provides a more natural sound experience compared to traditional RITE hearing aids, where externally placed microphones limit access to the natural acoustic modifications of the auricle and concha [16,25]. Similarly, the TIE design has been shown to improve speech intelligibility in both noisy and quiet environments and to achieve higher localization accuracy compared to RITE [1]. While subjective preference favored the standard receiver overall, a considerable proportion of participants (41.0%) preferred the M&RIE receiver, indicating that it provides meaningful benefits for certain users that outweigh the possible disadvantages of its relatively large size. Nevertheless, patients who preferred the standard receiver reported discomfort and pain when inserting the larger M&RIE receiver into the ear, highlighting the need for improvements in future versions of the device.

Subjective satisfaction, as measured by the APHAB questionnaire, showed no significant differences between the M&RIE and standard receivers across all subscales: EC, BN, RV, and AV. Interestingly, although not statistically significant, the M&RIE receiver demonstrated a slight trend toward better performance in background noise, suggesting potential advantages in noisy environments. This finding is consistent with the theoretical benefit of M&RIE technology, which is designed to improve spatial hearing and speech perception in complex listening situations by preserving natural pinna effects [6,26].

The audiological outcome, REIG, showed that the M&RIE receiver provided sufficient real-ear amplification, comparable to the standard receiver across all tested frequencies. This finding suggests that, despite concerns regarding potential amplification limitations due to the microphone’s proximity to the receiver, the M&RIE receiver effectively meets NAL-NL2 fitting targets without causing subjective discomfort. The absence of significant differences in REIG confirms that the M&RIE receiver does not compromise amplification performance and can be considered a reliable option for individuals with moderate hearing loss. These results are consistent with previous reports indicating that M&RIE devices maintain adequate amplification while preserving natural spatial cues [17]. However, it remains to be investigated whether speech recognition in noisy environments is better with the M&RIE receiver than the standard receiver.

A limitation of this study is the small sample size, which may be underpowered to detect subtle differences between the two receivers. Another limitation is that we did not consider the effect of different input levels in the real-ear gain measurement. Because the risk of feedback increases as input intensity rises, it is necessary to compare real-ear saturation responses between the two receivers. Further studies with larger sample sizes and more comprehensive hearing assessments across various input intensities are warranted to fully elucidate the benefits of M&RIE technology.

In conclusion, the M&RIE receiver demonstrated sufficient amplification, which was comparable to that of the standard receiver in terms of REIG, speech recognition, and user satisfaction.

## Figures and Tables

**Table 1:** Demographics of the enrolled patients (*n* = 39).

Characteristics	Value (%)
Age (yr)	72.4 ± 12.7 years
Sex	
Male	20 (51.3)
Female	19 (48.7)
First receiver	
Standard receiver	15 (38.5)
M&RIE receiver	24 (61.5)
Side of Hearing aid	
Bilateral	30 (76.9)
Unilateral	9 (23.1)
Hearing level (PTA_4_)	
Right	52.4 ± 11.1 dB HL
Left	48.9 ± 11.2 dB HL
Word recognition score at MCL	
Right	77.2 ± 13.8%
Left	74.4 ± 16.1%
Configuration	
Ski-slope type	30 ears (43.0)
Flat type	39 ears (57.0)
Receiver Preference	
Standard receiver	23 (59.0)
M&RIE receiver	16 (41.0)

## Data Availability

Data is contained within the article.
